# Efficacy of donepezil in patients with chronic tinnitus

**DOI:** 10.1016/j.clinsp.2026.101135

**Published:** 2026-09-09

**Authors:** Elaine Miwa Watanabe, Laura G.E. Vasconcelos, Maria F. Bonadia-Moraes, Fatima T. Husain, Nilo J.C. Duarte, Jeanne Oiticica

**Affiliations:** a Department of Otorhinolaryngology, Faculdade de Medicina da Universidade de São Paulo (FMUSP), São Paulo, SP, Brazil; b Department of Speech and Hearing Science, and the Beckman Institute for Advanced Science and Technology, University of Illinois Urbana-Champaign, Champaign, IL, USA; c Medical Investigation Laboratory (LIM-03), Faculdade de Medicina da Universidade de São Paulo (FMUSP), São Paulo, SP, Brazil

**Keywords:** Donepezil, Chronic tinnitus, Randomized clinical trial, Acetylcholinesterase, Double blind method, Neurotransmitters

## Abstract

**Introduction:**

Tinnitus is any perception of sound that occurs in the absence of an external auditory stimulus. Donepezil, commonly prescribed for dementia, has not yet been evaluated in individuals with tinnitus. By increasing synaptic availability of acetylcholine, a neurotransmitter widely distributed in the central nervous system and potentially neuroprotective against glutamate induced excitotoxicity, donepezil may offer a pharmacological approach for the management of this condition.

**Methodology:**

We conducted a double-blind, prospective, randomized controlled trial with a sample of 35 each in a chronic subjective, non-somatosensory tinnitus group and an age- and gender-matched control group. Participants received either donepezil or a placebo for 3-months. Treatment effects were monitored using scales and questionnaires: THI, VAS, CDR, MMSE; and psychoacoustic measurements of tinnitus.

**Results:**

No statistically significant difference in symptom improvement was observed between the donepezil and placebo groups as assessed by the evaluation instruments. Notably, analysis of confidence intervals for patients achieving a minimum improvement of ≥ 7 and ≥ 11-points in THI scores suggested a potential clinically meaningful trend favoring donepezil. Additionally, complete remission of tinnitus was observed in a single patient, occurring only in the donepezil group.

**Conclusion:**

In the present double-blind, randomized, placebo-controlled clinical trial, no statistically significant difference was identified between the groups regarding the primary outcome. However, the donepezil-treated group demonstrated better performance than the placebo group, and a possible indication of greater clinical benefit was observed in the treatment group in those using amplification, suggesting a potential therapeutic relevance, but larger, more in-depth studies are needed to confirm our findings.

## Highlights

• Donepezil has not been evaluated in patients with tinnitus.
• Donepezil may offer a pharmacological option for tinnitus management.
• THI responder analysis suggested a trend favoring donepezil.
• Treatment benefits appeared greater among amplification users.
• Complete tinnitus remission occurred only in the donepezil group.

## Introduction

Tinnitus is any perception of sound that occurs in the absence of an auditory stimulus. It affects 15% of the world population^1^ and 22% of the population in the city of São Paulo.^2^ One of the pathophysiological mechanisms of tinnitus is related to the increase in glutamate activity, the main excitatory neurotransmitter within the cochlea, which is also excitotoxic and capable of generating tinnitus.^3^^,^^4^ Although this glutamatergic toxicity is well established, the lack of a clearly effective drug for its treatment underscores the critical importance of studies investigating agents capable of reversing this process.^5^

The medication donepezil has not yet been studied in patients with tinnitus. It is a drug that inhibits the enzyme acetylcholinesterase, capable of increasing the synaptic availability of Acetylcholine (Ach). Ach is widely distributed in the central nervous system and plays an important role in cortical development and activity, learning, and memory.^6^ Donepezil also has a neuroprotective effect against glutamate-induced excitotoxicity and is habitually used to treat neurodegenerative diseases such as Alzheimer's, a disease in which acetylcholine is also decreased.^7^ According to some experimental studies, the mechanisms of neuroprotection of donepezil could be: 1) Neuronal protection against glutamatergic toxicity via the alpha-7 nicotinic Acetylcholine Receptor (alpha-7 nAChR) and via Src tyrosine kinase; 2) Reduction of caspase-3 induced by glutamate; 3) Attenuation of excess intracellular calcium influx caused by glutamate and 4) Reduction of the surface of glutamate receptors (N-Methyl-D-Aspartate ‒ NMDA) mediated via alpha-7 nAChRs.^8^^,^^9^

Furthermore, donepezil has also been associated with increased dopamine levels in the hippocampus, a region of the limbic system involved in depression and anxiety.^6^^,^^10^ These comorbidities have been frequently associated with tinnitus, suggesting that central regions involved in emotional and cognitive areas may contribute to its severity.^1^^,^^11^

Reports in the literature have also described cases of musical tinnitus remission with donepezil at a dose of 5 mg/day, and has even been considered by some authors as a first-line therapeutic option for this condition.^12^^,^^13^ A further advantage of this medication is its free availability in Brazil through the Unified Health System of Brazil (SUS).^14^

The objective of this double-blind randomized controlled trial or RCT, which provides the highest level of evidence regarding the efficacy of an intervention, is to compare the efficacy of donepezil with that of a placebo in patients with chronic subjective tinnitus.

## Material and methods

The study was approved on August 21, 2020, by the Ethics Committee for Research Project Analysis of the Hospital das Clínicas of the Faculty of Medicine of the University of São Paulo (CAPPesq) under number CAAE (Certificate of Presentation of Ethical Appreciation): 34251320.5.0000.0068, version 2, opinion 4.228.694.

### Study design

The study was designed as a prospective, placebo-controlled, randomized, double-blind clinical trial and conducted at the Tinnitus Research Group outpatient clinic, Department of Otorhinolaryngology, Faculty of Medicine, University of São Paulo (FMUSP) ‒ Brazil.

### Medication and placebo

The manufacture of the placebo, the masking of the active drug, and the randomization were performed by the pharmacy division of the Faculty of Medicine of USP. The donepezil drug used in this study was Don®, produced by Eurofarma Laboratory, a company responsible for supplying various medications to the Unified Health System (SUS) at an affordable cost to the general Brazilian population.^15^ The placebo was manufactured to identical in shape, size, and color with the drug, such that the patients or the dispensing researcher could not tell them apart.

### Intervention

The treatment consisted of 3-months under donepezil medication, starting with 1 tablet of 5 mg/day, for 30-days, followed by an increase in dose to 10 mg for another 60-days. The same dose regimen protocol is issued by the Ministry of Health for Alzheimer's disease.^14^ All patients randomized to the placebo group followed a similar protocol for the placebo pill during study and were subsequently treated with the active drug, after the completion of the study.

### Eligibility criteria

Inclusion criteria: Over 18-years old, with constant, unilateral or bilateral tinnitus, for more than 6-months, of sensorineural subtype (cochlear damage of any etiology, identified by audiometry and/or electrophysiological tests), without a somatic component.

Exclusion criteria: Pregnant women, lactating women, allergy or any known hypersensitivity to donepezil or piperidine derivatives, allergy to yellow dye, allergy or intolerance to lactose, patients with external and/or middle ear pathologies, tympanograms that are not type A, conductive hearing loss, supraventricular cardiac conduction abnormalities or bradycardia, history of seizure, schizophrenia, ulcer, severe asthma or COPD, patients using carbamazepine, dexamethasone, phenobarbital, phenytoin, rifampicin, ketoconazole, quinidine, medications analogous to donepezil (memantine, rivastigmine, galantamine) or suspended with less than 30-days of use.^15^

### Treatment evaluation

All patients underwent the following pre- and post-treatment evaluations with placebo or active drug, as described below:

• Clinical otorhinolaryngological assessment:

° To check any conditions that could be excluded from the group with sensorineural tinnitus.

° Education counseling: Both sets of participants received identical education counseling about tinnitus from an ENT physician who was blinded to their treatment status, during the screening and enrollment phase.

• Application of Treatment Evaluation Instruments:

° Visual Analogue Scale (VAS);^16^

° THI (Tinnitus Handicap Inventory);^17^

° MMSE (Mini-Mental State Examination);^18^

° CDR Scale (Clinical Dementia Rating Scale).^19^

• Measurement of psychoacoustic characteristics of tinnitus (acufenometry): Pitch and loudness matching and identification of Minimum Masking Levels (MML) were performed by during the audiological assessment, at the audiology service of the Clinical Audiology Outpatient Clinic of the Faculty of Medicine of the University of São Paulo.

• Donepezil plasma concentrations were determined by Liquid Chromatography-Mass Spectrometry (LC-MS) and were made feasible by the Biochemistry Laboratory of the Hospital das Clínicas of the University of São Paulo (DLC-HCFMUSP). Method validation was approved in accordance with the guidelines and directives defined by the Quality Control Commission, which complies with the regulations and reference legislation adopted by the Central Laboratory Division of the Hospital das Clínicas of the Faculty of Medicine of the University of São Paulo (DLC-HCFMUSP), allowing the use of the TSQ-ALTIS Thermo Scientific® equipment for the analysis of donepezil plasma concentrations (Chalom MY et al., 2020).^20^ For this study, specific Donepezil calibrators from Toronto Research Chemicals (TRC, Vaughn, Ontario, Canada) were imported.

### Pilot project and sample size calculation

Before beginning the RCT, we conducted a feasibility study. Based on this pilot study of 9 cases, an average reduction in THI with the use of donepezil (3-months treatment, 5 mg for 30-days followed by 10 mg for 60-days) of 12-points with a variability of 11 points (SD = 11 points) was observed. Assuming a reduction of at least 10 points in THI (considering the study by Zeman et al. (2013),^21^ a clinically significant improvement in tinnitus treatment is at least 7 points), with 80% power and 95% confidence, the necessary sample for the study was determined to be 19 subjects in each group. The calculation was based on the two-tailed Student's *t*-test.

### Statistical analysis

Qualitative characteristics were described for the groups using absolute and relative frequencies and the association between groups was verified using Chi-Square tests or exact tests (Fisher's exact test or likelihood ratio test). The demographic variables were described using summary measures (mean, standard deviation, median, and quartiles) and compared between groups using unpaired Student's *t*-test and Mann-Whitney test, respectively.^22^

The outcome measures were described separately for the groups at the two assessment timepoints and compared using Generalized Estimating Equations (GEE) with normal distribution with identity or logarithmic link function, according to the Kolmogorov-Smirnov normality test of data distribution, assuming an AR(1) correlation matrix between evaluation moments,^23^ with differences estimated using Bonferroni multiple comparisons.^24^ Among excluded cases the reasons for exclusions were described according to group and the association was verified using Fisher's exact tests.^22^

The IBM-SPSS for Windows version 22.0 software was used for the analyses and Microsoft Excel 2013 software was used for data tabulation. Tests were performed with a significance level of 5%. For the analysis of confidence intervals (95%), Jamovi version 2.3 and R Core Team version 4.1 software were used.

## Results

### Randomized selection

**Fig. 1:**
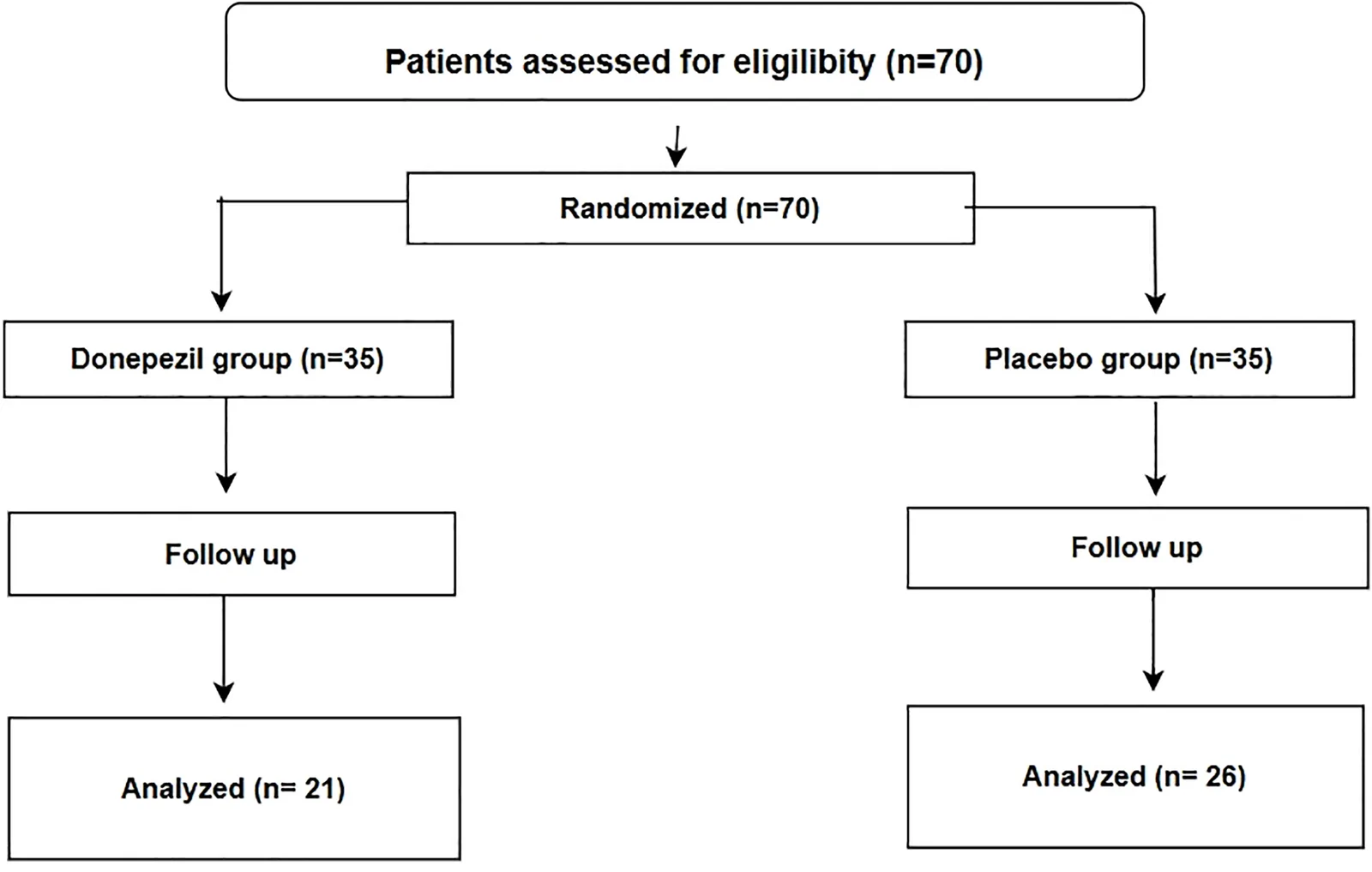
Flowchart of patient enrollment.

The admission of 70 patients to the study began on November 19, 2021, and final data collection was completed on October 11, 2024. The randomized allocation of eligible patients was 35 patients for the donepezil group and 35 patients for the placebo group, as shown in Fig. 1.

### Loss to follow-up (Dropout)

After the start of treatment, 14 individuals (40%) in the active drug group dropped out, 13 due to side effects (93%) and 1 (7%) due to change of address. In the placebo group, 9 individuals (25.7%) were excluded: 5 due to side effects (55.6%), 3 (33%) due to irregular use of medication, and 1 (11%) due to personal reasons (recovery from orthopedic surgery).

It was observed that among the reasons for dropout, adverse effects were predominantly higher in the donepezil group compared to the placebo group (p = 0.006).

Additionally, three individuals allocated to the placebo group were excluded from the analysis due to the detection of false information, identified after unblinding. Blood dosage alone would not be able to detect eventual dissimulation in the placebo group. Therefore, all volunteers were asked to return the bottles at the end of treatment under the pretext of “appropriate disposal of the bottles”. And although these 3 patients had stated that they completed the treatment, the bottles were returned with more than 30 tablets. Thus, after exclusion, the data from these patients did not compromise the statistical analysis of this study.

### Dropout analysis by adverse effects

Although the adverse effect was the main reason for treatment discontinuation, especially in the active drug group, when considering each of these undesirable reactions separately, it was not possible to identify any significant association between the groups (p > 0.05).

### Incidence of adverse effects

As demonstrated in Table 1, the active group displayed a higher incidence of cases than the placebo group. However, the complaint of “dry mouth” was reported only in the placebo group (2 participants), as shown in Table 1.

**Table 1:** Distribution of adverse effects.

Adverse effects	Donepezil (n = 35) n (%)	Placebo (n = 35) n (%)
Insomnia	5 (14.3)	0 (0)
Nausea	3 (8.6)	0 (0)
Epigastralgia	3 (8.6)	1 (2.9)
Inappetence	2 (5.7)	1 (2.9)
Nightmare	2 (5.7)	0 (0)
Dizziness	1 (2.9)	2 (5.7)
Headache	1 (2.9)	1 (2.9)
Tremors	1 (2.9)	1 (2.9)
Restlessness	1 (2.9)	1 (2.9)
Chills	1 (2.9)	0 (0)
Diarrhea	1 (2.9)	0 (0)
Fatigue	1 (2.9)	0 (0)
Itching	1 (2.9)	0 (0)
Cramp	1 (2.9)	0 (0)
Dry mouth^a^	0 (0)	2 (5.7)

a Occurred only in the placebo group.

### Participants

Of the total of 70 patients selected (35 in the donepezil group and 35 in the placebo group), 47 patients (67.1%) completed the treatment, 24 (51%) were female and 23 (49%) were male, with ages between 41- and 77-years (mean of 59-years). Of these 47 individuals, 21 were allocated to the donepezil group and 26 to the placebo group; hearing aid use was present in 7 and 6 participants, respectively. All patients resided in the city of São Paulo. Both groups showed statistically similar personal characteristics of sex, age and hearing aid use.

### Psychoacoustic profile of tinnitus pre-treatment

In both studied groups, the psychoacoustic characteristics of tinnitus were statistically similar (p > 0.05) in terms of hissing or whistling sound perception (p = 0.851); bilateral, right or left laterality (p = 0.310); pitch (p = 0.784), loudness (p = 0.992), and MML (p = 0.09), as shown in Table 2.

**Table 2:** Psychoacoustic profile of tinnitus.

Variable	Group		Total (n = 47)	р
	Donepezil (n = 21)	Placebo (n = 26)		
Tinnitus sound perception, n (%)				0.851
Hissing	14 (66.7)	18 (69.2)	32 (68.1)	
Whistling	7 (33.3)	8 (30.8)	15 (31.9)	
Laterality of tinnitus, n (%)				0.310
Right-sided	4 (19)	10 (38.5)	14 (29.8)	
Left-sided	6 (28.6)	7 (269)	13 (27.7)	
Bilateral	11 (52.4)	9 (34.6)	20 (42.6)	
Pitch (kHz)				0.784
Mean ± SD	6.7 ± 4.1	6.6 ± 3.9		
Loudness (dBSL)				0.992
Mean ± SD	7.3 ± 6.3	7.8 ± 3.8		
MML (dBSL)				0.09
Mean ± SD	7 ± 4.4	8.6 ± 5.8		

Chi-square test.

### Comparison of pre- and post-treatment psychoacoustic characteristics

There was no statistically significant difference in pitch, loudness, or MML at either pre and post-treatment assessments in both groups (p > 0.05), but there was a trend for improvement in MMLs for the Donepezil group, as shown in Table 3.

**Table 3:** Pre- and post-treatment psychoacoustic characteristics.

Variable	Placebo		Donepezil		p_group	p_time	p_interaction
	Pre	Post	Pre	Post			
Pitch (khz)					0.784	0.841	0.294
Mean ± SD	6.6 ± 3.9	7.2 ± 3.5	6.7 ± 4.1	6.6 ± 3.8			
Loudness (dBSL)					0.992	0.642	0.353
Mean ± SD	7.8 ± 3.8	7.2 + 3.5	7.3 ± 6.3	7.4 ± 6.4			
MML (dBSL)					0.090	0.217	0.586
Mean ± SD	8.6 ± 5.8	8.4 ± 4.5	7 + 4.4	5.6 ± 2.9			

GEE with normal distribution and identity link function.

### Treatment evaluation

General analysis of donepezil and placebo groups, time, and interaction to evaluate the intervention, VAS, THI, CDR scale, and MMSE scores were compared for main effects of group, time, and their interactions (Table 4):

• p(group): comparison of whether there is a difference between the placebo and active drug groups.
• p(time): comparison of whether there is a difference between pre and post-intervention moments.
• p(interaction): verifies whether the behavior of the studied groups was similar over the evaluated moments.

**Table 4:** Evaluation instruments according to group, time, and interaction.

Variable	Placebo		Donepezil		p_group	p_time	p_interaction
	Pre	Post	Pre	Post			
VAS					0.811	0.234	0.234
Mean ± SD	6.8 ± 1.8	6.8 ± 2	7.1 ± 1.7	6.2 ± 2.9			
THI					0.762	0.045	0.387
Mean ± SD	44.6 ± 23.6	42.5 ± 23.8	44.3 ± 19.9	38.9 ± 24.5			
THI Functional					0.945	0.034	0.927
Mean ± SD	18.5 ± 12	16.5 ± 11.4	18.7 ± 9.1	16.8 ± 11.3			
THI Emocional					0.785	0.119	0.355
Mean ± SD	15.7 ± 9.8	15.2 ± 9.9	15.6 ± 9.2	13.8 ± 9.7			
THI Catastrophic					0.200	0.263	0.077
Mean ± SD	10.4 ± 3.6	10.8 ± 3.8	10±3	8.3 ± 6.3			
CDR^a^					0.550	0.030	0.627
Mean ± SD	0.37 ± 0.5	0.17 ± 0.34	0.33 ± 0.37	0.1 ± 0.21			
MMSE					0.729	0.398	0.081
Mean ± SD	27.3 ± 3.3	27.9 ± 3.4	28 ± 2.3	27.8 ± 3			

GEE with normal distribution and identity link function.

a GEE with normal distribution and logarithmic link function; For all analyses, a first-order autoregressive correlation matrix [AR(1)] was assumed between time points.

There was no statistically significant difference between groups (p_group > 0.05) for the VAS scale (p_group = 0.811), THI Questionnaire (total THI p_group = 0.762; functional THI p_group = 0.945; emotional THI p-group = 0.785; catastrophic THI p_group = 0.200), CDR (p_group = 0.555), and MMSE (p_group = 0.729).

Although total THI (p_time = 0.045), functional THI (p_time = 0.034), and CDR (p_time = 0.030) stand out for being the only ones to have shown a difference between pre- and post-treatment moments (p_time < 0.05), this was demonstrated in both groups: donepezil and placebo. The post-hoc analysis showed a statistical power of 42% for the comparison between time points.

Thus, when analyzing the behavior of the two groups pre and post-treatment, the p_interaction was > 0.05 in all presented variables, further underscoring that there was no significant difference between the groups.

**Fig. 2:**
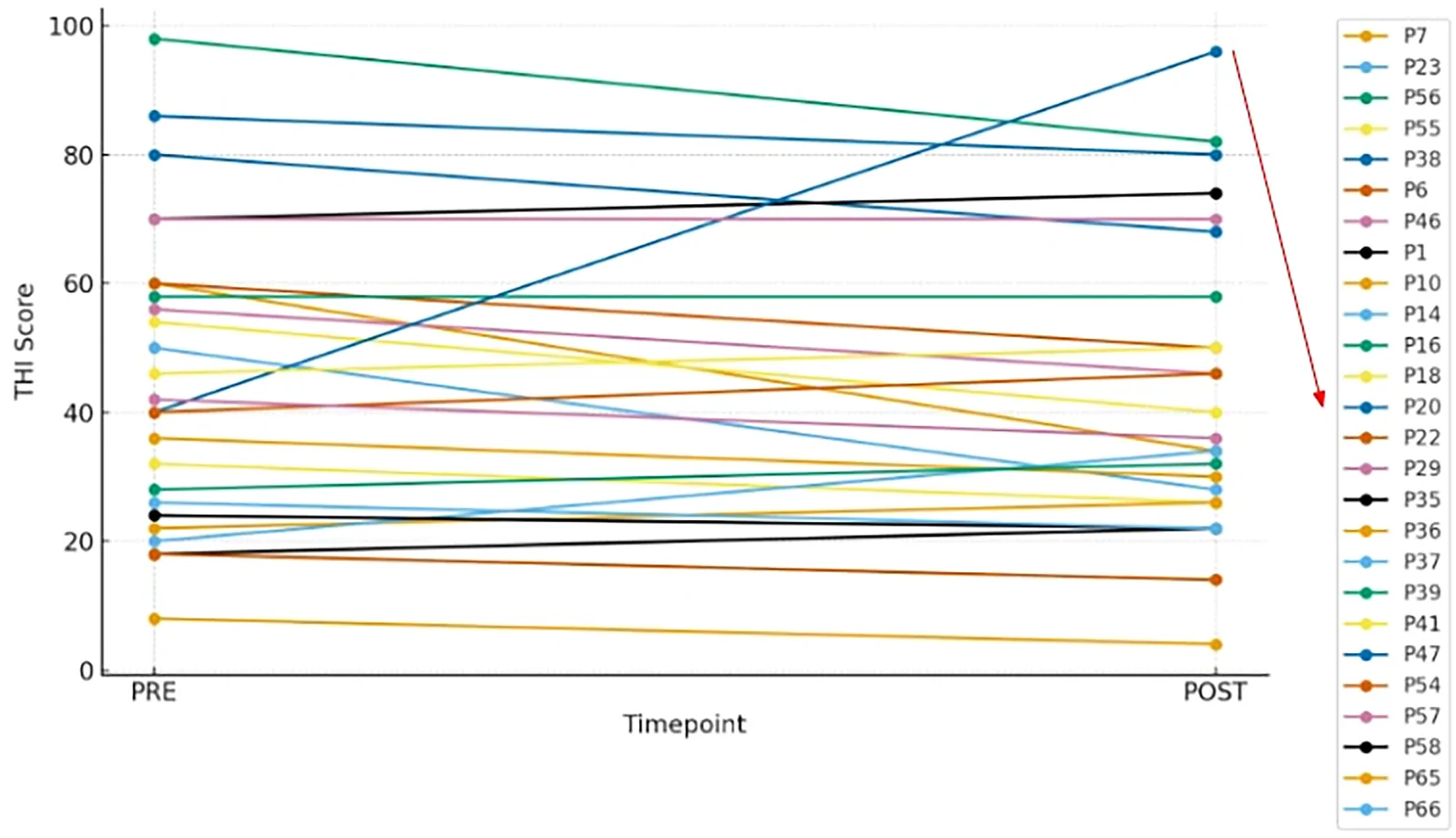
Individual THI scores at placebo group pre and post treatment. Note: red arrow pointing to the worst case in terms of increase in THI scores.

**Fig. 3:**
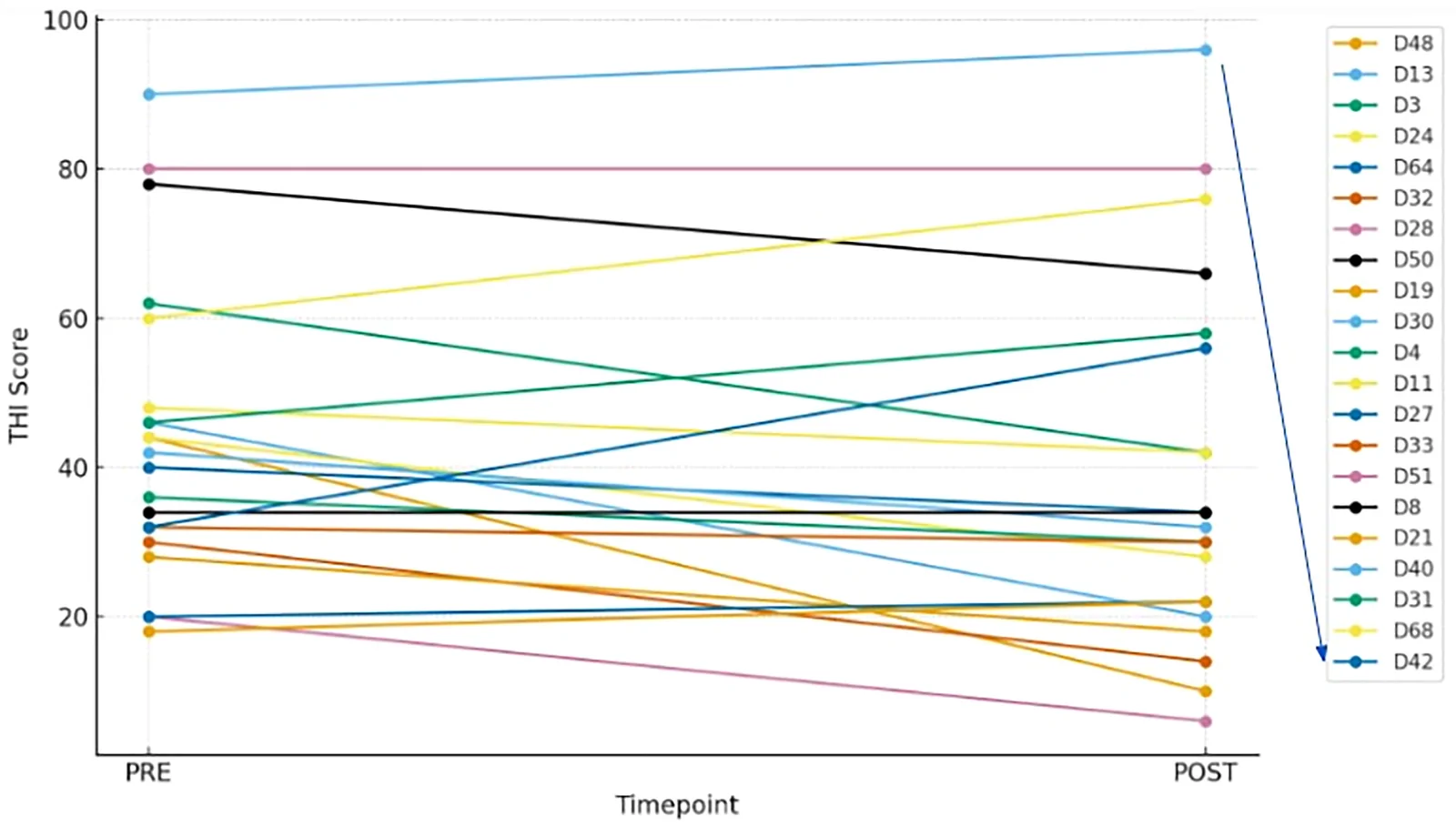
Individual THI scores at donepezil group pre and post treatment. Note: Blue arrow pointing to the worst case in terms of increase in THI scores.

Considering the evolution of THI scores in both groups, the worst-case outcome in the donepezil group was still better than that observed in the placebo group. Fig. 2, Fig. 3 illustrate the individual THI evaluations for the placebo and donepezil groups, respectively, pre and post treatment. In Fig. 2, the red arrow indicates the worst case in the placebo group (ID P20), who got 56-points worse. In Fig. 3, the blue arrow indicates the participant with the worst outcome in the Donepezil group (ID D42), who exhibited a worsening of 34-points.

Analysis of the subgroup that improved with THI ≥ 7 points and VAS ≥ 2 points THI values ≥ 7 and VAS ≥ 2 points are considered indicative of clinically significant improvement in patients with tinnitus:^21^^,^^25^ With respect to this study, of the 21 patients in the donepezil group, 9 (42.9%) showed improvement in THI ≥ 7 and 6 (28.6%) in VAS ≥ 2. At the same time, of the 26 participants in the placebo group, 7 (26.9%) had THI ≥ 7 and 4 (15.4%) in VAS ≥ 2.

Although improvement was more frequent in the donepezil group, the difference was not statistically significant for either VAS ≥ 2 (p = 0.306) or THI ≥ 7 (p = 0.252), and the post-hoc analysis showed low statistical power (31%).

### Analysis of confidence intervals

To enhance the robustness of the analysis of cases with THI improvement ≥ 7 points, confidence interval estimation was adopted. To ensure comparability between groups, the confidence intervals were first analyzed based on the THI scores obtained in the pre-treatment period for both the donepezil and placebo groups. As shown in Table 5, with a 95% Confidence Interval (95% CI), the mean initial THI was 44.3 points in the donepezil group and 44.6 in the placebo group.

**Table 5:** Pre-treatment THI values in donepezil and placebo groups.

Initial THI	Group	
	Donepezil (n = 21)	Placebo (n = 26)
Mean	44.3	44.6
Mean lower bound (95% CI)	35.2	35.1
Mean upper limit (95% CI)	53.3	54.1
Standard Deviation	19.9	23.6
Minimun value	18	8
Maximum value	90	98

Note: The 95% CI for the mean assumes a *t*-distribution with N-1 degrees of freedom.

Subsequently, when specifically considering cases that showed an improvement equal to or greater than 7 points in THI (Table 6), the mean THI score among responders was lower in the donepezil group (26.2, 95% CI 11.9–40.6) compared with the placebo group (49.7, 95% CI 32–67.4). Although the confidence intervals showed partial overlap, the mean difference between groups was 23.5 points, favoring donepezil. The estimated standardized effect size was large (Cohen’s *d* ≈ 1.24), suggesting a substantial magnitude of difference between the groups.

**Table 6:** Post-treatment THI values in donepezil and placebo groups that improved ≥ 7 points.

Final THI	Group	
	Donepezil (n = 9)	Placebo (n = 07)
Mean	26.2	49.7
Mean lower bound (95% CI)	11.9	32
Mean upper limit (95% CI)	40.6	67.4
Standard Deviation	18.7	19.2
Minimun value	6	28
Maximum value	66	82

Note: The 95% CI for the mean assumes a *t*-distribution with N-1 degrees of freedom.

### Supplementary analysis

When the study was designed, the accepted Minimal Clinically Important Difference (MCID) value to show treatment improvement was a decrease in THI ≥7 points. However, a recently published paper^25^ suggests that for a longer-term treatment such as ours, the metric should be a change in THI score of 11 points at 12 weeks of treatment, which is closer to our 90 days or three months of intervention.

We re-analyzed the data with the new MCID metric of ≥ 11 points. We found that 19% of those taking the placebo drug showed improvement, whereas 33% of those taking donepezil showed improvement with the new metric. Although the difference did not reach statistical significance (p = 0.270) and the post-hoc analysis showed a low statistical power (29%), an exploratory analysis of the confidence intervals (Table 7) also suggested a possible indication of greater improvement in the donepezil group.

**Table 7:** Post-treatment THI values in donepezil and placebo groups that improved ≥ 11 points.

Final THI	Group	
	Donepezil (n = 7)	Placebo (n = 05)
Mean	26.6	50.4
Mean lower bound (95% CI)	6.98	21.4
Mean upper limit (95% CI)	46.2	79.4
Standard Deviation	21.2	23.4
Minimun value	6	28
Maximum value	66	82

Note: The 95% CI for the mean assumes a *t*-distribution with N-1 degrees of freedom.

The mean post-treatment THI score was lower in the donepezil group (26.6) than in the placebo group (50.4), corresponding to an approximate difference of 23.8 points, favoring donepezil. Despite the partial overlap of the confidence intervals and the limited sample size, the magnitude of this difference suggests potential clinical relevance. In addition, the estimated effect size was large (Cohen’s *d* ≈ 1.08).

## Discussion

### Main findings

This RCT sought to determine the efficacy of donepezil compared to placebo. The descriptive statistical analysis of the sample demonstrated homogeneity in the population characteristics of the individuals and in the psychoacoustic profile of tinnitus, all with chronic, subjective, non-pulsatile and non-somatosensory tinnitus. Thus, such aspects did not demonstrate any statistically significant influence on the final outcome. At the end of the intervention, no statistically significant differences were observed in the change in tinnitus handicap in either group, i.e., both groups showed similar decrease in their handicap on average.

No serious adverse events were observed during treatment, supporting the safety of the intervention within the predefined inclusion and exclusion criteria. Although the donepezil group exhibited a higher frequency of adverse events, no statistically significant difference between groups was detected overall. Notably, the increased dropout rate in the donepezil group was largely attributable to adverse events. This differential dropout may represent a potential source of attrition bias and may have affected treatment effect estimation, thereby limiting the accurate identification of actual responders to the intervention.

In the donepezil-treated group, a higher frequency of participants reported insomnia and nausea. Indeed, the only complaint that exceeded the information described in the Don® drug monograph as “common reaction between 1 and 10%”,^15^ was the incidence of insomnia (14.3% of cases). Additionally, two cases of “dry mouth” (5.7%) were reported exclusively in the placebo group, an effect not described in the leaflet provided with the study. All other side effects presented in both groups were described in the official drug literature.

Regarding treatment adherence, confirmation through objective measurement of blood concentrations provided a highly reliable indicator, minimizing the likelihood of outlier values in the statistical analysis. There was 100% adherence in the donepezil group and 85.7% in the placebo group. That is, all patients who admitted to having undergone treatment in the donepezil group actually did so, confirmed by blood dosage of the medication.

### Placebo effects

Having a placebo group in clinical trials is necessary to accurately the effects of an intervention and the real magnitude of a treatment. This is particularly so in the case of tinnitus. A systematic review and meta-analysis of RCTs in tinnitus, aiming to ascertain the relevance of the placebo effect, demonstrated the positive impact of a placebo, providing relief and improvement in quality of life.^26^

In an older study,^27^ an improvement of 25% or more was observed in 40% of patients with tinnitus treated with placebo. However, the metrics currently used such as VAS and/or THI questionnaire were not used at the time, but rather a subjective scale in which the patient scored an improvement or worsening on a scale from 0 to 100.

Thus, placebo treatment has been shown to improve patient-reported tinnitus outcomes on standardized measures such as the THI and VAS-L; however, the improvement is not as substantial as nonplacebo treatment.^27^ This is mirrored in the findings of the present study. A substantial improvement was observed in the placebo group, with 26.9% of participants demonstrating a ≥ 7-point reduction in THI scores and 19% demonstrating a ≥ 11-point reduction, although proportionally lower than in the active treatment group.

The improvement observed in the placebo group may be partly explained by placebo-related effects, including patient expectations and conditioning mechanisms, which are particularly relevant in subjective conditions such as tinnitus. In addition, regular follow-up and increased clinical attention may have contributed to non-specific therapeutic effects.

### Clinical relevance

Although there was a significant improvement in total THI, functional THI, and CDR, in the pre and post-treatment moments, this behavior occurred similarly in both groups: active drug and placebo. Therefore, when performing the interaction analysis, comparing the behavior of both groups, it was not possible to say that this treatment actually improved chronic sensorineural tinnitus.

Given that this medication enhances cognitive function ‒ increased acetylcholine availability is associated with improvements in memory and learning ‒ an improvement in CDR performance and in the functional domain of the THI would be expected, as was observed.

This study did not reproduce the 12-point improvement in THI observed in the pilot study during the post-treatment period. Because the primary outcome did not reach statistical significance, additional post hoc exploratory responder analyses were performed based on clinically meaningful THI improvement thresholds (≥7 and ≥11 points). In these analyses, the donepezil group showed a greater reduction in tinnitus-related handicap compared with placebo.

A higher proportion of participants met the responder thresholds in the donepezil group than in the placebo group. For the ≥7-point threshold, improvement was observed in 42.9% of participants receiving donepezil compared with 26.9% in the placebo group. Similarly, for the ≥ 11-point threshold, improvement occurred in 33% of the donepezil group and 19% of the placebo group. Although the confidence intervals partially overlapped in both exploratory analyses (Tables 6 and 7), the observed differences favored the active treatment, with mean reductions of approximately 23.5 and 23.8 points in THI for the ≥ 7-point and ≥ 11-point responder thresholds, respectively.

The estimated effect sizes were large (Cohen’s *d* ≈ 1.20 for the ≥ 7-point group and 1.08 for the ≥ 11-point group), suggesting a potentially clinically meaningful benefit. In addition, the mean post-treatment THI scores in the donepezil group were 26.2 and 26.6 for the ≥ 7-point and ≥ 11-point responder groups, respectively, corresponding to a mild Tinnitus Handicap (THI 18–36). In contrast, the mean post-treatment THI scores in the placebo group were 49.7 and 50.4, respectively, corresponding to a moderate level of handicap (THI 38–56).^17^

Given the relatively small sample size and the resulting wide confidence intervals, these findings should be interpreted with caution. Although these analyses do not allow definitive conclusions regarding the efficacy of donepezil, they suggest a possible signal of clinical benefit that warrants confirmation in larger randomized trials.

### Case studies

Some specific particularities in this study drew attention and the clinical significance may perhaps be further explored in future studies.

A single case of total remission of tinnitus was observed in a 75-year-old male patient, with an improvement of 34 points in THI, after treatment with donepezil. When the patient underwent audiological testing, it was not possible to measure the tinnitus as it was absent, even in an attempt to retest after waiting for one hour. However, two days after the suspension of the medication at the end of the study, tinnitus reappeared. Subsequently, when the patient resumed the medication, at the same dosage, the patient noticed that the tinnitus disappeared again. No other factors, change in habit, or medication that could explain this phenomenon were identified. The only changes reported by the patient during the intervention period were the death of his father and financial difficulties, factors that could plausibly have exacerbated tinnitus rather than improved it.

Another case was that of a participant who showed an improvement of 10 dBSL of hearing at the exact frequency of the tinnitus pitch. And the other, although it improved 10 dBSL in hearing, was at a close frequency (6 KHz), but not identical frequency to the tinnitus pitch (8 KHz). Both participants showed an improvement of ≥ 7 points in THI, with reductions of 16 and 10 points, respectively. None of these participants used hearing aids, and no other factors were identified that could explain such audiometric changes. It is also noteworthy that these audiometric findings were not observed in any other patient who reported a reduction of ≥ 7 points in THI in the placebo group.

The donepezil group demonstrated a trend for MML improvement in comparison to the placebo group. Furthermore, the worst case occurred in the placebo group, in which the THI worsened by 56 points (from 40 to 96), whereas the worst case in the donepezil group worsened by 34 points (from 32 to 56), indicating that, in this regard, there were poorer outcomes in the placebo group.

Notably, the three patients with the greatest improvement in the active group were utilizing amplification devices, whereas no patients in the placebo improvement group were. This suggests that donepezil combined with amplification may lead to a better outcome. Hearing loss is a recognized risk factor for the development of tinnitus and cognitive impairment. It is plausible that improving memory and learning functions due to the increase in acetylcholine levels, contributes to the neuromodulation of tinnitus, consequently leading to its amelioration. This may further contribute to explaining why patients with musical tinnitus, who tend to use hearing aids, have benefited from treatment with donepezil or similar acetylcholinesterase inhibitors in reported cases.^12^^,^^13^ And if it is definitively confirmed that donepezil can contribute to a minimum auditory recovery of minimum 10 dBSL at the tinnitus pitch, as has been observed in the two patients in our study, this finding may justify the clinical improvement experienced by certain patients.

Some studies suggest potential benefits of donepezil on peripheral pathways involved in neurocortical reorganization in the context of hearing loss and auditory processing, given that acetylcholine acts as a neuromodulator in the inferior colliculus within the auditory pathway.^28^^,^^29^ Additionally, other studies have reported a reduction in P300 latency in the auditory pathway following treatment with donepezil (5 mg for one month), indicating a possible improvement in auditory cognitive processing.^30^^,^^31^ This neuromodulatory effect may help to explain the observed responses in a subset of patients in the treatment group, but not in the placebo group.

Nevertheless, these findings should be interpreted cautiously given the small number of participants.

### Caveats and future directions

A major caveat of this study is the small sample size resulting from attrition, largely attributable to adverse events, particularly in the donepezil group, thereby limiting the robustness of the conclusions. However, there was a trend towards improvement in the donepezil group overall and particularly so in several cases.

Considering the clinical aspects of these participants in the treatment group who showed remarkable improvement, and focusing on the drug's mechanisms concerning the improvement of memory and cognition areas via acetylcholine, as well as a possible glutamatergic excitotoxic activity,^6^^,^^7^ future studies may be able to better explore the changes observed in this clinical trial. We observed that those showing an improvement in the tinnitus handicap, tended to use hearing aids, and experienced hearing improvement at the tinnitus pitch as well as their minimum masking level. Therefore, future studies should pre-define subgroups based on hearing aid use, laterality, or psychoacoustic profile.

Our clinical findings can serve to generate hypotheses about the mechanism of donepezil’s therapeutic response and the subgroups of patients who may most benefit from such treatment. Studies with larger sample sizes are needed to confirm these findings and better stratify responders from non-responders.

## Conclusion

In the present double-blind, randomized, placebo-controlled clinical trial, no statistically significant difference was identified between the groups regarding the primary outcome. However, the donepezil-treated group demonstrated better performance than the placebo group, and a possible indication of greater clinical benefit was observed in the treatment group in those using amplification, suggesting a potential therapeutic relevance, but larger, more in-depth studies are needed to confirm our findings.

## Data availability

All data supporting the findings of this study are included within the article.

### IA declaration

During the preparation of this work the author(s) used ChatGPT in order to assist in generating the plotting code for Fig. 1. After using this tool, the authors reviewed and edited the content as needed and take full responsibility for the content of the published article.

### Authors’ contributions

Elaine Miwa Watanabe: Conceptualization; data curation; investigation; writing-original draft.

Laura G. E. Vasconcellos: Audiological assessments.

Maria F. Bonadia-Moraes: Audiological assessments.

Fatima T. Husain: Review editing.

Nilo J. C. Duarte: Clinical analysis of blood test results.

Jeanne Oiticica: Supervision and Funding acquisition

## Declaration of competing interest

The authors declare no have conflicts of interest. The funders had no role in the design of the study; in the writing of the manuscript, or in the decision to publish.
